# Evaluation of the Antimalarial Effect of *Ferulago angulata* (Schlecht.) Boiss. Extract and Suberosin Epoxide Against *Plasmodium berghei* in Comparison with Chloroquine Using *in-vivo* Test

**Published:** 2016

**Authors:** Seyed Ebrahim Sajjadi, Nader Pestechian, Mahnaz Kazemi, Mohammad-Ali Mohaghegh, Ahmad Hosseini-Safa

**Affiliations:** a *Department of Pharmacognosy, School of Pharmacy, Isfahan University of Medical Sciences, Isfahan, Iran. *; b *Department of Parasitology, School of Medicine, Isfahan University of Medical Sciences, Isfahan, Iran.*; c *Isfahan Pharmaceutical Sciences Research Center, Isfahan University of Medical Sciences, Isfahan, Iran.*

**Keywords:** *Ferulago angulate*, Suberosin epoxide, *Plasmodium berghei*, *Antimalaria*

## Abstract

Resistance to most antimalarial drugs has encouraged the development of novel drugs. An alternative source for discovering such drugs is natural products. Some *Ferulago* species are used in folk medicine for their sedative, tonic and anti-parasitic effects. Besides, coumarins isolated from this genus found to have in vitro anti-leishmanicidal effect. The present study is aimed to evaluate the *in-vivo* antimalarial activity of *Ferulago angulata* (Schlecht.) Boiss. extract and suberosin epoxide, using suarian mice. A rodent malaria parasite, *Plasmodium berghei* was used to inoculate healthy male Swiss Albino mice of age 6-8 weeks and weight 23-27 g. Hydro-alcoholic extract of *F. angulata *(20, 100, 300, 600 mg/Kg) and suberosin epoxide suspension (10, 30, 50, 100 mg/Kg) were administered subcutaneously. Parameters including percentage of parasitemia, suppression of parasitemia and mean survival time were determined using standard test such as peter^٬^s.

Chemo-protective effects were exerted by the crude extract and suberosin epoxide. Maximum effect was observed with the larger doses of the crude extract and suberosin epoxide. Suberosin epoxide increased the survival time compared to chloroquine. However, the results of this study indicate that the plant has a promising anti-plasmodial activity against *plasmodium berghei*. Thus, it could be considered as a potential source of new antimalarial agents. Suberosin epoxide at the dose of 100 mg/Kg possesses relatively significant antimalarial effect. Chemical derivatization of the parent compound or preparation of the modified formulation is required to improve its systemic bioavailability.

## Introduction

 Malaria is one the most prevalent, widespread parasitic infectious disease in the world, which causes approximately 0.7-1 million deaths per year. Nearly half of the world population is at the risk of malaria (1, 2). Most of the cases (78%) occur in the African region, Southeast Asia (15%) and eastern Mediterranean regions (5%) (3). Drug resistant of plasmodium falciparum have been found in many endemic areas of the world and many of conventional antimalarial drugs have been associated with treatment failure. The disease is endemic in Iran with 16000 cases in 2008 (4). Despite intensive efforts to control malaria, the disease continues to be one of the greatest health problems in south eastern part of the country. In Iran P. falciparum resistance to chloroquine has been reported since 1983 (5). Due to the increasing resistance of the parasite to available drugs, development of new agents is one of the main ways to overcome this issue. Antimalarial drug development can be done by minor modifications of existing agents or designing novel agents (6).

Natural products provide relatively cheap treatment opportunities for malarial infections. Quinine and artemisin derivatives, the two most important currently available antimalarial agents are from natural sources. In case of artemisin, chemical modifications of the natural parent compound have led to the synthesis of a series of highly potent antimalarial agents (7, 8). The development of these two important drugs from natural sources and the utilization of many plants traditionally in various parts of the world triggered the search for new antimalarial drugs of natural origin using *in vitro* and *in-vivo* studies.

*Ferulago angulata* (Umbelliferea) is a native plant, commonly found on mountains of west of Iran. Other than Iran it also grows in Turkey, Greece and Serbia (9, 10).

*F. angulata* is used traditionally in Iran as a decreasing agent of the blood sugar and as a food additive to prevent food spoiling. Recently studies revealed antioxidant and anti-tumor effect against leukemia (11, 12).

**Figure 1:**
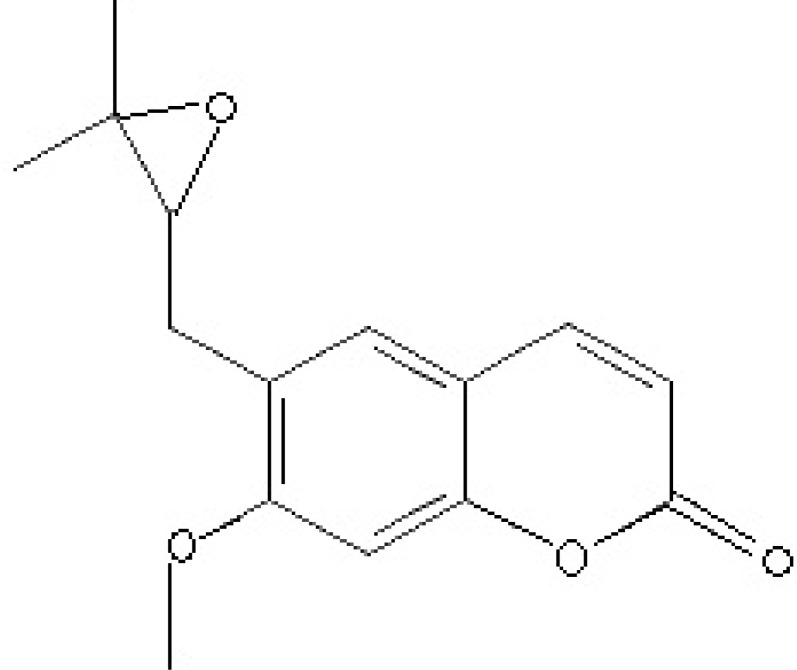
Suberosin epoxide

Coumarins are benzo-α-pyrone derivatives which have a wide range of activities including anti-inflammatory, antioxidant and anti-HIV activities (14, 15). Nervous system protection and anti-leishmanial effects are also the reported effects of some of coumarins (15-17). Based on the previously observed anti-parasitic effects mentioned above, the present study evaluated the in vivo antimalarial activity of the crude extract of *F. angulata* and suberosin epoxide (Figure 1.) as one of its main coumarin content*.*

## Experimental

*Materials and Methods*

*Selection and collection of plant material*

Fresh aerial parts of *F. angulata* were collected May 2013 from Yasuj in Kohgiluye-Boyerahmad province in the west of Iran. The fresh leaves were wrapped with plastic sheets during transportation. The plant was identified as *F. angulata* by a taxonomist and a herbarium specimen was deposited (No. 1138) at the School of Pharmacy and Pharmaceutical Sciences, Isfahan University of Medical Sciences, Isfahan, Iran for future reference.

*Extraction*

The aerial parts of *F. angulata* were air dried at room temperature under shade and reduced to appropriate size by grinding with an electric mill. Dried plant material (300 g) were extracted by maceration using ethanol:water (80:20) as solvent for 24 h. The extraction process was facilitated using an orbital shaker at 120 rpm. The mixture was first filtered using Whatman paper filters. The residue was re-macerated for 1 h and filtered again. Re-maceration and filtration was repeated one more time. The ethanol part of the extract was then retrieved by a rotary evaporator (Heidolph, Germany). The remaining part of the extract was kept in the freezer at -20 °C and then it was dried using freeze dryer. The dried extract was kept at −20 °C until use.

200 g of the grinded powder of *Ferulago angulata* aerial parts were put into a filter paper and placed into the Soxhlet. Then 300 mL n-hexane was added and the heater was turned on to complete the extraction. The n-hexane extract was concentrated after 20 times of reflux. The concentrated extract was placed into the refrigerator. Primary crystals of cumarin appeared after 2 days. Primary elution was done by cold n-hexane. Recrystallization with pure n-hexane was done 4 times for refining coumarin crystals.

 Purity of the crystals was tested using TLC, heptane-ethyl acetate (70-30) as mobile phase and sodium sulfate-molibdate as indicator. Suberosin epoxide isolated from *F. angulata *was used as main component of coumarin fraction (18).

*In-vivo antimalarial tests*

*Animals and parasite*

 Male Swiss albino mice (6–8 weeks age, weighting 23–27 g) bred and maintained at the Razi Research Institute were used. They were kept under standard condition (temperature of 22 ± 3 °C, relative humidity of 40-50% and 12 h light/12 h dark cycle), fed with food and water in the animal house of School of Medicine, Isfahan University of Medical Sciences, Isfahan, Iran. Animals were kept for three days for adaptation to the experimental environment. Chloroquine sensitive strain of *Plasmodium berghei* (ANKA) obtained from the Department of Parasitology, School of Medicine, Isfahan, Iran was used. The parasites were maintained by serial passage of blood from infected mice to non-infected ones on weekly basis.

*Parasite inoculation*

Albino mice previously infected with *P. berghei* and parasitemia level of 20% were used as donor. The donor mice were then sacrificed and blood was collected by cardiac puncture into heparinized falcon tube. The blood was then diluted with physiological saline (0.9%) based on parasitemia level of the donor mice and the red blood cell (RBC) count of normal mice (19), in such a way that 1 mL blood contains 5 × 10^6 ^infected RBCs. Each mouse was then given 0.2 mL of this diluted blood intraperitoneally, which contained 1 × 10^6 ^*P. berghei* infected RBCs.

*Grouping and dosing of animals*

For evaluating of the extract and suberosin epoxide, infected mice were randomly divided into ten groups of 8 mice per group. Groups of 1 to 4 were treated with the crude extract of *F. angulata* at 20 mg/Kg (CF20), 100 mg/Kg (CF100), 300 mg/Kg (CF300) and 600 mg/Kg (CF600), respectively. 

Groups of 5 to 8 were treated with suberosin epoxide at 10 mg/Kg (SE10), 30 mg/Kg (SE30), 50 mg/Kg (SE50) and 100 mg/Kg (SE100). 

The remaining two groups were also served as positive and negative controls and administered chloroquine 20 mg/Kg (CQ20) and 2% tween 80 diluted in physiological saline (CON), respectively (20). The volume administered in each case was 0.2 mL.

*The 4 day suppressive test*

This test was used to evaluate the schizontocidal activity of the extract and the fractions against *P. berghei* infected mice according to the method described by Peter *et al*. (21). Treatment was started three hours after mice had been inoculated with the parasite on day 0 and then continued daily for four days from day 0 to day 3. After completing the treatment, thin blood film was prepared from the tail of each animal on day 4, 7, 14 and 21 to determine parasitemia and percentage of inhibition. Each mouse was also daily observed for determination of survival time.

*Parasitemia measurement*

Thin smears of blood were made from the tail of each mouse on day 4, 7, 14 and 21 for Peter’s test. The smears were applied on microscope slides (76 × 26 mm), fixed with absolute methanol, stained with 10% Geimsa stain, then washed gently using distilled water and dried at room temperature. Stained slides for each mouse were examined under Olympus microscope (CH30RF200, Japan) with an oil immersion using 100× magnification. Ten different fields on each slide were examined to calculate the average parasitemia as shown below (22).

$\mathrm{\% parasitemia}=\frac{\mathrm{Number of parasited RBC}}{\mathrm{Total RBC number}}\times 100$

Finally, percent parasitemia suppression was compared with control and parasitemia suppression was calculated using the following formula (23).

$\mathrm{\% suppression}=\frac{\mathrm{mean parasitemia of negative control}-\mathrm{mean parasitemia of treated group}}{\mathrm{mean parasitemia of negative control}}\times 100$

*Data analysis*

Windows SPSS Version 19.0 was used to perform statistical analysis. The data are presented as means ± standard error of mean (SEM). The results were analyzed statistically using one-way ANOVA and two-tailed student᾽s t-test to identify the differences between treated groups and control. P-Value of less than 0.05 was considered statistically significant.

**Figure 2:**
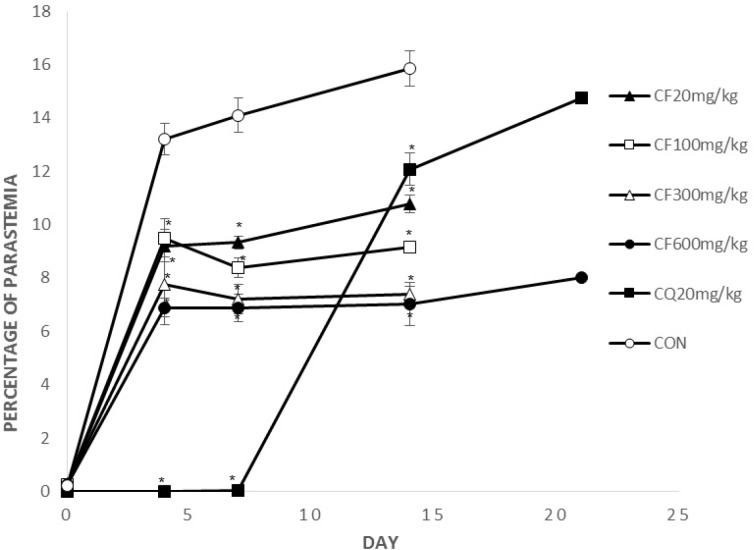
Mean parasitemia of the treated groups with crude extract on the day 0, 4, 7, 14, 21

Data are expressed as mean ± SEM; CQ = chloroquine, CON = Control, CF= crude extract of* Ferulago angulata*. Numbers refer to dose in mg/kg. * = P<0.05 compared with the control groups.

**Figure 3:**
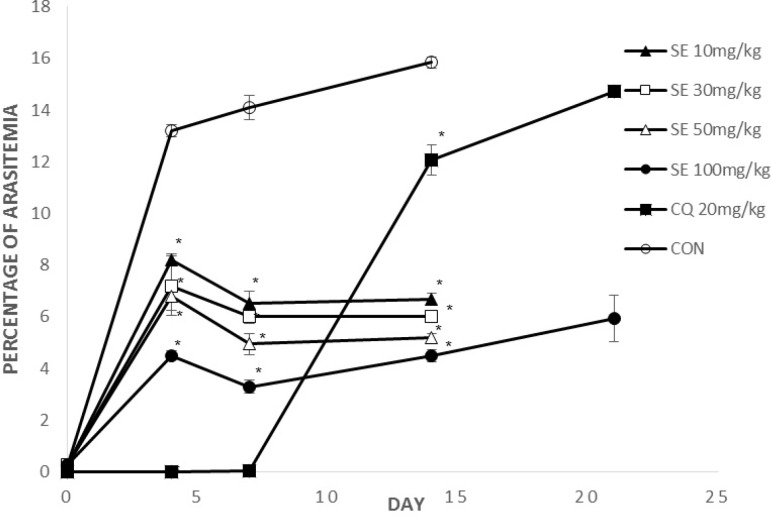
Mean parasitemia of the treated groups with suberosin epoxide on the day 0, 4, 7, 14, 21

Data are expressed as mean ± SEM; CQ = chloroquine, CON = Control, SE= suberosin epoxide. Numbers refer to dose in mg/kg. * = P<0.05 compared with the control groups.

**Figure 4:**
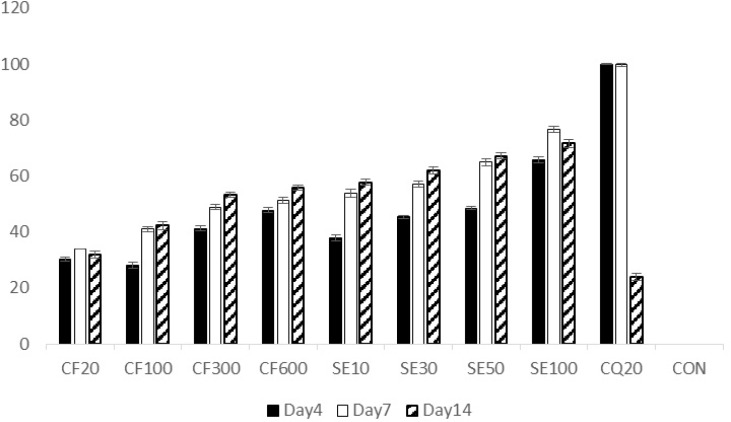
Percentage of suppression of infected mice treated with crude extract and suberosin epoxide of *Ferulago angulata* in the 4 day suppressive test.

CF= Crude extract of *Ferulago angulata*, SE= suberosin epoxide. Numbers refer to dose in mg/kg. P<0.05 in all groups compared to negative control group.

**Figure 5:**
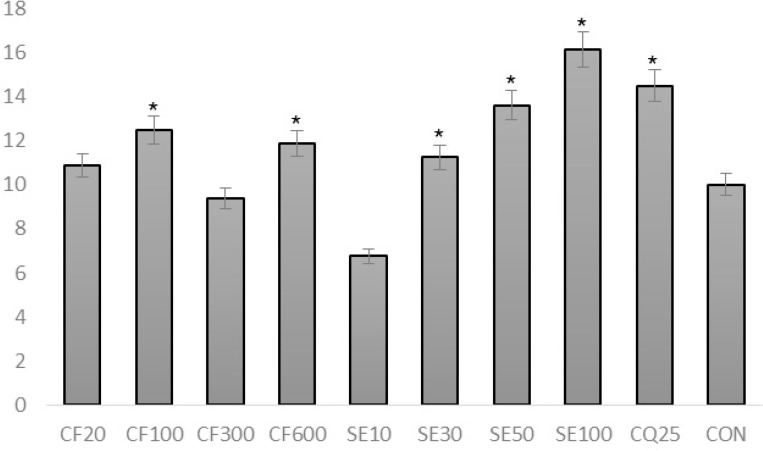
Survival time (day)

CF= Crude extract of *Ferulago angulata*, SE= suberosin epoxide. Numbers refer to dose in mg/kg.

* = P<0.05 compared with the control groups.

## Results

Antimalarial activity of the extract and suberosin epoxide was evaluated by comparing the parasitemia (%), parasitemia suppression (%) and mean survival time of the treated groups in comparison to the negative control group.

Figure 2, 3 show parasitemia (%) on day 0, 4, 7, 14, 21. 

On days 4 and 7, average parasitemia of the groups treated with crude extract of *F. angulata* and suberosine epoxide was significantly (p < 0.05) lower than that of negative control group.

However, none of the observed activities of the extracts and suberosin epoxide was comparable to that of chloroquine (p >0.05).

On day 14, average parasitemia of all groups (CF, SE, CQ) was significantly lower than negative control group. The observed activity of the extract (CF100, 300, 600) and suberosin epoxide (SE10, 30, 50, 100) was comparable (p < 0.05) to that of chloroquine.

Remaining mice by the day 21 were those treated with CQ20, CF600 and SE100. Parasitemia of these remaining groups on day 21 is shown in Figure 2, 3.

Parasitemia of these two remaining groups was significantly (p < 0.05) lower than chloroquine.

Initial parasite suppression (%) of mices treated with chloroquine (100%) and the groups treated with exctract and suberosin epoxide, was significantly higher than the negative control group (0%) (p<0.05 in all cases) (Figure 4.). However, by the day 14, parasite suppression of the extract (CF100, 300, 600) and suberosine epoxide (SE10, 30, 50, 100) were significantly (p > 0.05) higher than chloroquine.

Suberosin epoxide (ranging from 30 mg/Kg to 100 mg/kg) was capable of significantly increasing the survival time compared to negative control group (p<0.05) (Figure 5.). 

SE100 possessed the most significant effect of increasing mean survival time among all groups including positive control group (CQ20) (p<0.05). 

## Discussion

Coumarin, a class of plants secondary metabolites, has been reported to have a wide range of pharmacological properties including antiplasmodial activity in different antimalarial assays (24, 25). Adesanwo *et al.* have reported the anti-malarial effect of a coumarin called eniotorin from the root bark of* Quassia undulate*, in which the aqueous extract and the isolated compounds exhibited dose-related effect against the *P. falciparum* malaria parasite in an *in-vitro* antimalarial assay (26).

It has been demonstrated by Argotte *et al*. that the extract of the stem bark of *Hintonia latiflora* showed the suppression of total parasitemia and the chemo suppression of schizont numbers, when tested *in vivo* against *P. berghei* infection in mice. Bioassay-directed fractionation of the extract, using the *in-vitro* 16 h and the in vivo 4-day suppression tests on *P. berghei* schizont numbers were done. New compounds, 5-O-beta-D-glucopyranosyl-7,4′-dimethoxy-3′-hydroxy-4-phenylcoumarin (1), along with the known 5-O-beta-D-glucopyranosyl-7-methoxy-3′,4′-dihydroxy-4-phenylcoumarin (2) were isolated from the extract and tested for the possible antimalarial effect. These two compounds suppressed the development of *P. berghei* schizonts *in-vitro*. Compound 2 suppressed the development of schizonts at the dose of 40 mg/kg in the *in-vivo* assay (27). These results are in accordance with ours. However, the in vitro test has not been used in our study and mean survival time has not been evaluated in the studies mentioned above. 

In this survey anti-plasmodial activity of *F.angulata* extract and suberosin epoxide as its major coumarin, was studied. There are some reports on antiplasmodial activity of plants used in Iranian traditional medicine (29) and this plant has also been used as an anti-parasitic agent (18). Treatment with extract and suberosin epoxide significantly inhibited parasitemia of *P.berghei* infection in swiss albino mice compared to negative control, implying direct parasiticidal activity. 

The four day suppressive test, which mainly evaluates the antimalarial activity of candidates on early infections, has been done for antimalarial drug screening. *P. berghei*, a rodent malaria parasite, has been used as an appropriate model for accurate comparison of the *in-vivo* activity of drugs (28). *In-vivo* model has been chosen since the possible prodrug effect and possible involvement of the immune system in eradication of infection would affect the results (19). 

Based on the results of the in-vivo antimalarial testing, suberosin epoxide, which is the main coumarin of the plant, exhibited promising antimalarial activity with regards to its inhibitory activity on the reduction of parasitemia and the prolongation of survival time. However, suberosin epoxide at the dose of 10 mg/kg didn᾽t have significant effect on survival time. 

It may be also possible to consider the plant as a potential source of antimalrial agents. Although the active compound is yet to be identified, the antimalarial activity of *F. angulata* could be attributed to a single or a combination of its secondary metabolites such as coumarins. Some of these metabolites have been reported to have antimalarial activity (24, 25, 27).

## Conclusions

The present study indicates that crude extract of *F. angulata* and suberosin epoxide have antiplasmodial activity. Suberosin epoxide appeared to be superior in supressing parsitemia and enhancing survival time. The findings suggest that coumarins are probably responsible of antimalarial activity of the plant. However, future studies on the plant regarding antimalarial activity should be conducted using crude extract or suberosine epoxide beside chloroquine to examine the synergistic effect of co administration of these compounds. Further studies such as the prophylactic test and curative test (Rane test) are required.
